# Case report:^18^F-2-deoxy-2-fluoro-D-glucose positron emission tomography/computed tomography image findings of a dog with gossypiboma

**DOI:** 10.3389/fvets.2023.1107238

**Published:** 2023-08-03

**Authors:** Su-Hyeon Kim, Sungin Lee

**Affiliations:** Department of Veterinary Surgery, College of Veterinary Medicine, Chungbuk National University, Cheongju-si, Chungbuk, Republic of Korea

**Keywords:** gossypiboma, ^18^F-deoxy-2-D-glucose, positron emission tomography/computed tomography, dog, canine

## Abstract

A 13-year-old, spayed, female mixed breed dog that had previously undergone mastectomy and ovariohysterectomy at our institution was referred to the nuclear medicine department for metastasis evaluation following surgery. ^18^F-deoxy-2-D-glucose positron emission tomography/computed tomography (18F-FDG PET-CT) was performed and a soft-tissue mass was observed in the abdominal cavity. The characteristics of the abdominal mass were assessed and screening for metastasis was done with follow-up 18F-FDG PET scans. Uptake of ^18^F-deoxy-2-D-glucose was higher in the peripheral region and lower in the center of the abdominal mass. Exploratory laparotomy was performed, and the removed abdominal mass was consistent with a gossypiboma, which is a retained surgical sponge composed of non-absorbable material with cotton matrix. This case report describes the characteristics of 18F-FDG PET-CT imaging in a dog with an abdominal gossypiboma, which has not been reported in the veterinary literature before.

## 1. Introduction

Gossypiboma, derived from the Latin *gossypium*, which means cotton and the Swahili word *boma:* place of concealment, is a rare condition in veterinary medicine, and its diagnosis can be challenging (1). The characteristics of gossypibomas on radiography, ultrasonography, and computed tomography (CT) have been reported in the veterinary literature (2, 3). However, little is known about ^18^F-fluorodeoxyglucose (^18^F-FDG) positron emission tomography (PET) features of gossypibomas in a dog. In human medicine, positron emission tomography (PET)/computed tomography (CT) using ^18^F-2FDG has been used to detect an abdominal gossypiboma (4, 5) with a hallmark: a lesion of gossypiboma shows low central FDG uptake with high peripheral FDG uptake. This case report describes ^18^F-FDG PET/CT features of an abdominal gossypiboma in a dog who had previously undergone abdominal surgery.

To our best knowledge, abdominal gossypiboma in a canine, identified using PET/CT, has not been reported in the veterinary literature. Therefore, this is the first report describing the 18F-FDG findings in a clinical case of a dog with gossypiboma.

## 2. Case description

A 13-year-old, spayed, female mixed breed dog was referred initially to the nuclear medicine department for metastasis evaluation after surgery at our institution. The dog displayed anorexia, with normal heart rate, respiratory rate, rectal temperature, and Doppler-induced systolic blood pressure. A complete blood (cell) count (CBC), serum biochemical analysis, and electrolyte analysis showed normal values for all parameters except elevations of alanine aminotransferase (751 U/l; reference range, 21–102 U/l), alkaline phosphatase (991 U/l; reference range, 0–97 U/l). Two months prior to this presentation, the patient was diagnosed with a mammary gland tumor after which total mastectomy and ovariohysterectomy were performed. Histopathologic examination of the resected mammary gland mass revealed a characteristic mammary comedocarcinoma with a mitotic count >20 per 10 high power fields (HPFs) and incomplete surgical margins (IDEXX Laboratories, Inc., USA). Different modalities were performed to evaluate possible distant metastasis after surgery, including 18F-FDG PET/CT (PET/CT; Discovery-72 STE, General Electric Medical Systems, Waukesha, WI, 73 USA).

**Figure 1:**
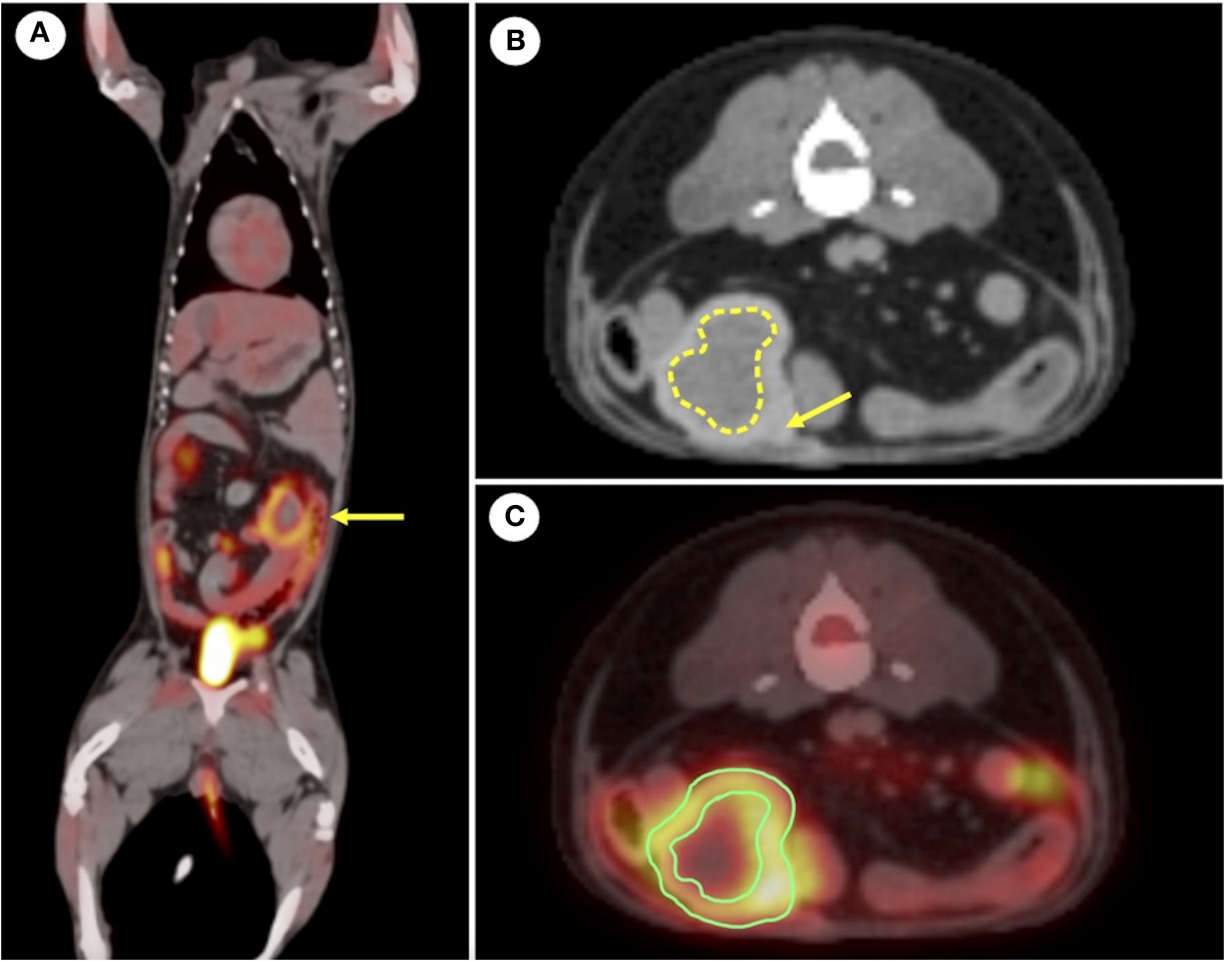
**(A)** Fused positron emission tomography-computed tomography (PET-CT) images using 18F-2-deoxy-2-fluoro-D-glucose (18. F-FDG). Increased 18F-fluorodeoxyglucose uptake in the peripheral region of the abdominal mass (yellow arrow) is observed in dorsal plane. **(B)** Representative computed tomography image of postcontrast. The yellow arrow indicates the peripheral region that is hyperattenuating. The dotted line indicates the central region that is hypoattenuating. **(C)** Region of the interest (ROI) was drawn in the transverse plane for the abdominal mass. Maximal standardized uptake value (SUV) is 4.747 and mean SUV is 2.590. Contrast enhancement is seen in the peripheral region of the abdominal mass.

The patient was put on fasting for at least 12 h before scanning. Blood glucose assessed just prior to the PET scan was normal (104 mg/dL; reference range 81–133 mg/dL). The dog was premedicated with midazolam (0.2 mg/kg; Midazolam, Bukwang Pharm. Pharm. Co., Ltd., Seoul, South Korea) after intravenous catheterization and placement of an indwelling urinary catheter. Anesthesia was induced with intravenous propofol (6 mg/kg; Provive, Myungmoon Pharm. Co., Ltd., Seoul, South Korea), and endotracheal intubation was performed. Under general anesthesia maintained with isoflurane (Terrell, Piramal Critical Care, Bethlehem, PA, USA), the dog was ventilated with 100% oxygen and was infused with Hartmann's solution (5 mL/kg/h) IV. After induction of anesthesia, the dog was positioned in sternal recumbency, and the position was maintained throughout CT and PET scans. CT images (pre- and post-contrast) were obtained prior to the PET scan (Discovery-STE, General Electric Medical Systems, Waukesha, WI, USA). The CT images were obtained using a Muti-detector CT scanner with 100 mAs, at 120 kVp in 1.25 mm slice thickness. Contrast scans were performed with intravenous administration of 880 mgI/kg iohexol (Omnipaque, GE Healthcare, Marlborough, MA, USA). Diagnostic CT scans were acquired in the resting expiratory position. The pre-contrast CT scan showed a soft tissue mass in the abdominal cavity with homogeneous hypoattenuation of the central region and hyperattenuation of the peripheral region. The post-contrast CT scan showed contrast enhancement at the mass periphery. PET/CT scan was performed after intravenous administration of 0.53 mCi (0.17 mCi/kg) 18 F-FDG, followed by a 45-min uptake period. Emission scans were obtained as static frames for about 2 min per bed position, with five bed positions. The average time between CT and PET images was approximately an hour. CT and PET scans can be obtained with a single device. The average total anesthesia time for CT and PET imaging was approximately 3 h. The PET images were analyzed using a commercial program (OsiriX MD v10.0; Pixmeo Sarl, Geneva, Switzerland). The peripheral region of the suspected abdominal mass showed a high level of 18F-FDG uptake (Figure 1). The regions of interest were drawn manually on the PET/CT fusion images. The standardized uptake value (SUV) is a measurement that normalizes tissue 18F-FDG uptake (MBq/mL) relative to the injected dose (MBq) per body weight (g). Therefore, SUV was measured in the region of the abdominal mass suspected to be a gossypiboma, which showed increased 18F-FDG uptake. The calculated maximum and mean SUVs of the abdominal mass were 4.472 and 2.590, respectively. The abdominal mass, which visually revealed higher 18F-FDG uptake in the peripheral region and lower uptake in the center, was confirmed to have inflammation or tumor infiltration aspects. The patient seems to be required regular metastasis evaluation so that radiography and ultrasonography were performed for further follow-up evaluation to set the reference point. And they also used to assess the suspected abdominal mass.

**Figure 2:**
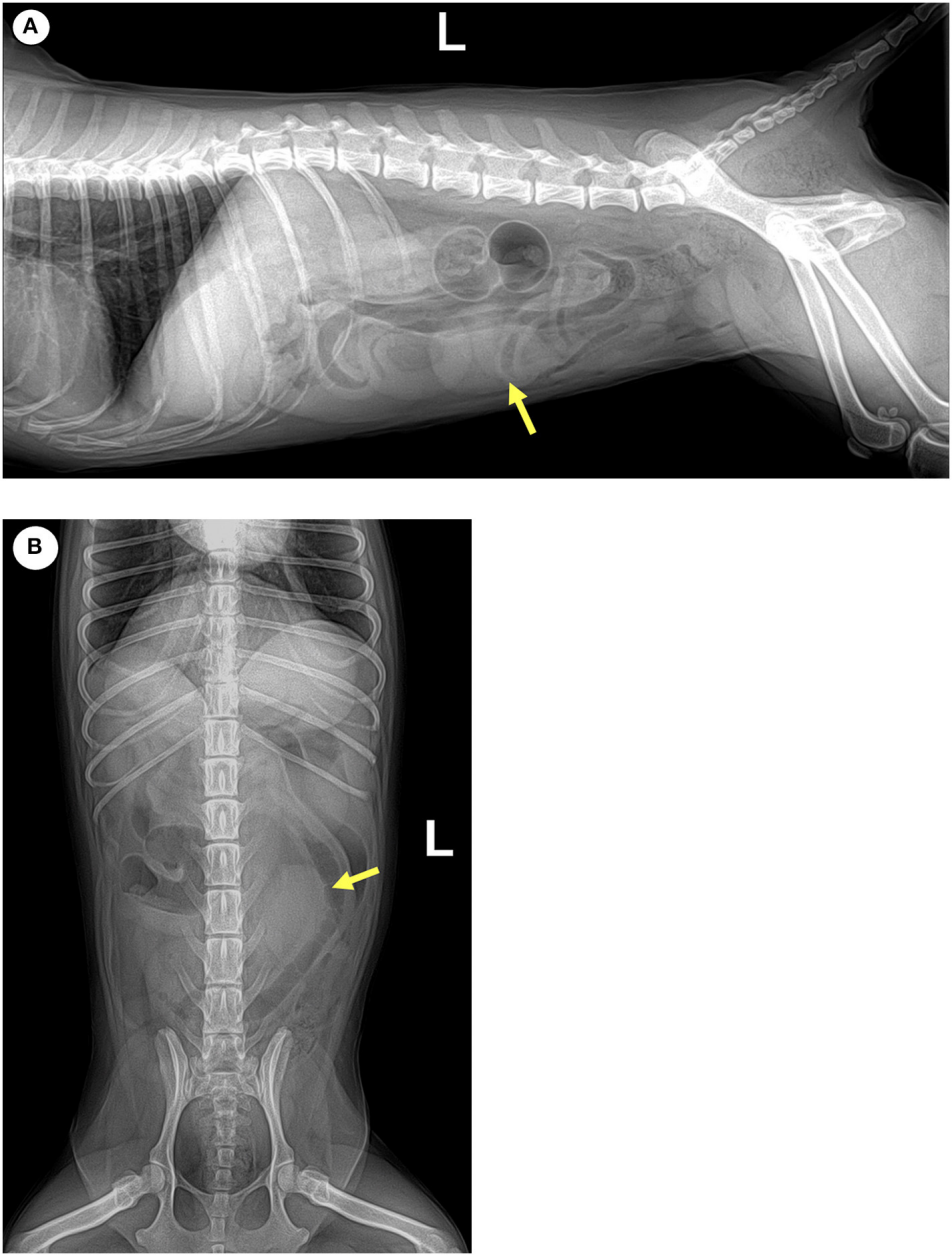
Yellow arrows indicate an ovoid, well-defined, soft tissue opque mass on **(A)** left lateral and **(B)** ventrodorsal radiographs of the caudal portion of the abdomen.

**Figure 3:**
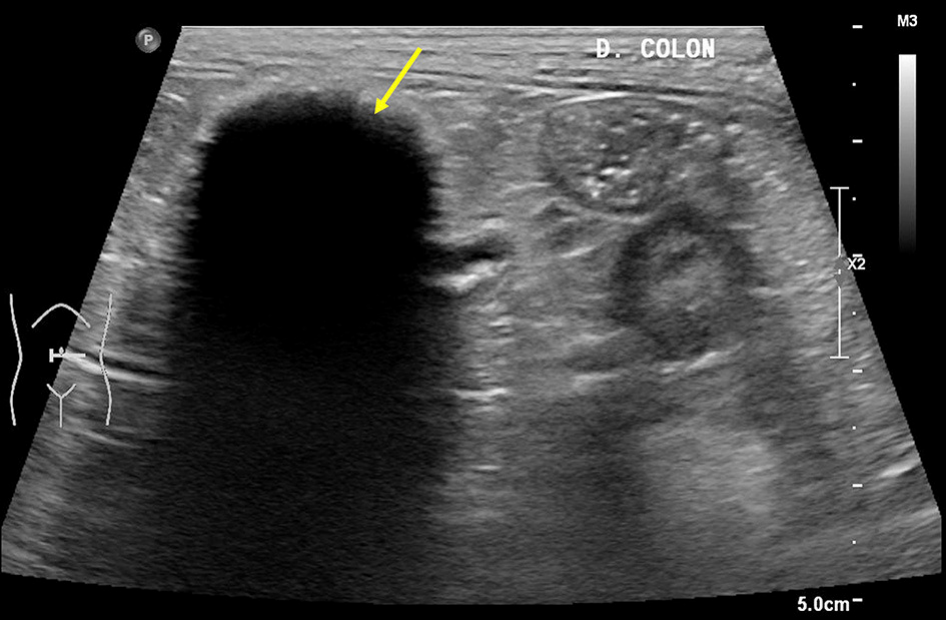
Transverse ultrasonographic view of the mass-like structure at the medial part of the descending colon. Yellow arrow indicates no evidence of continuity to the gastrointestinal tract with echogenic stripes associated with strong acoustic shadowing. On a color Doppler scan, no vascularization or twinkling artifacts were discovered.

Radiographs of the abdomen showed a 2.76 x 3.76 cm radiopaque mass at the left caudoventral region of the abdominal cavity (Figure 2). The mass was well-defined, round-to-oval shape and with soft tissue opacity, which did not show connectivity to surrounding abdominal organs. Abdominal ultrasonography revealed a mass-like structure in the ventral region of the abdomen, which was likely to be in the medial part of the descending colon. The mass lesion appeared compact and round with a strong acoustic shadow and irregular margins. No vascularization or twinkling artifacts were found on a color Doppler examination. No obvious continuity to the gastrointestinal tract was observed (Figure 3). Given the basis of imaging appearances and clinical history, a diagnosis of an inflammatory process, such as secondary to a chronic foreign body like gossypibomas was suspected.

**Figure 4:**
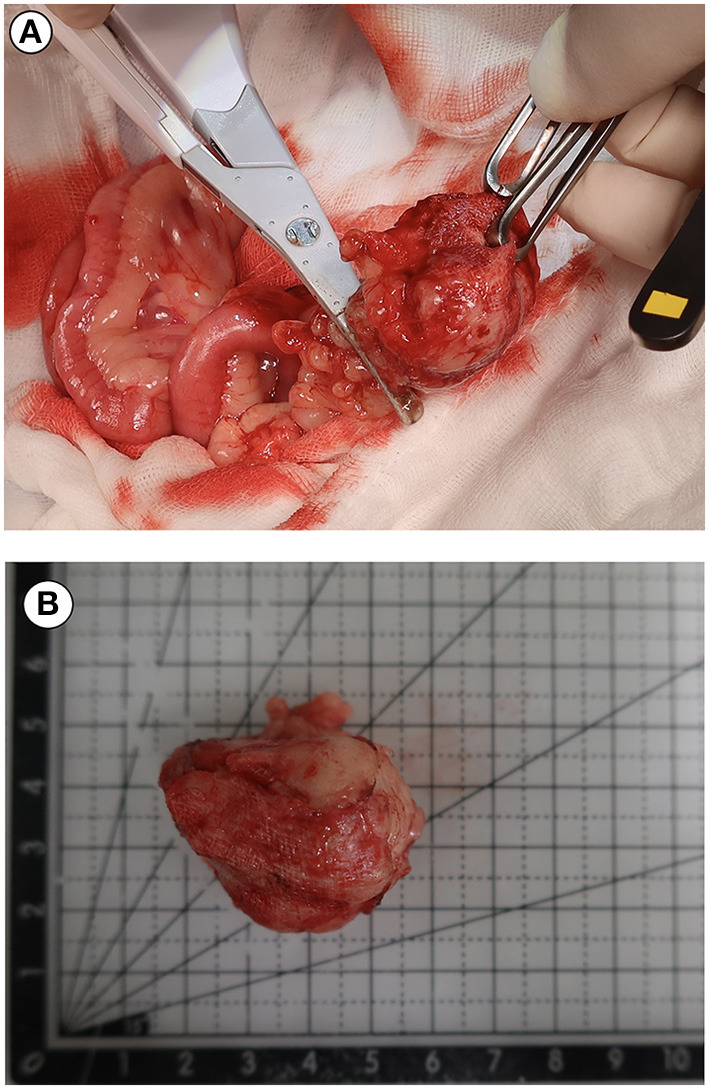
Intraoperative photograph during exploratory laparotomy **(A)** and retained gauze photograph **(B)** after sectioning of the mass. The mass was proved to contain a surgical sponge consistent with a diagnosis of gossypiboma.

We considered that the abdominal mass-like structure could be a foreign body material such as a retained surgical sponge or a possible result of inflammation. Therefore, an exploratory laparotomy was planned under general anesthesia maintained by propofol intravenously (10 mg/kg; Provive, Myungmoon Pharm. Co., Ltd., Seoul, South Korea) following midazolam (0.2 mg/kg; Midazolam, Bukwang Pharm. Pharm. Co., Ltd., Seoul, South Korea) premedication. The patient was secured in the dorsal recumbent position and the abdomen was opened with a midline incision. Grossly, the mass-like structure appeared yellow and thick, located between small intestine loops. The textile material with a foreign body reaction in the surrounding soft tissues was removed by dissection from adhesions to the small intestine and mesentery. No remarkable findings were observed during the remaining exploratory laparotomy. Before closing the abdomen, all precautions and count of surgical sponges were taken. The patient recovered successfully from the anesthesia. The mass retracted from the abdominal cavity was removed and cut open. Its composition and structure conformed as surgical sponges, which was consistent with gossypiboma (Figure 4).

The patient was discharged 6 days postoperatively and showed no significant clinical signs. One month after retraction of the surgical sponge, the dog remained asymptomatic. No further follow-up for evaluating metastasis was performed because the owner declined it.

## 3. Discussion

The clinical signs of gossypiboma can vary from asymptomatic to fatal (6). The immune system can show two responses to foreign bodies: exudative or fibrous type (2). The exudative type, characterized by abscess formation or chronic internal or external fistula formation, occurs relatively soon after surgery and can be discovered early. Fibrous type reaction, as seen in this case, results in granuloma formation, adhesions, and encapsulation. The patient though asymptomatic, developed characteristic fibrous type reaction, and the retained surgical gauze was incidentally detected (6). Diagnosing gossypibomas based on clinical signs is difficult. Therefore, knowledge of the imaging characteristics of gossypibomas is important. Radiography, ultrasound, CT, and magnetic resonance imaging are useful tools for detecting retained sponges (1). In human medicine, ^18^F-FDG PET/CT has also been used for identifying and diagnosing gossypibomas (7).

Various imaging modalities including X-ray, ultrasound, CT and PET/CT were used in this case to evaluate the patient for metastasis, which led to the detection of a retained surgical gauze. Radiographs are actually the most commonly used method to detect gossypibomas (8, 9). Common appearance of retained surgical gauge includes the presence of incorporate radiopaque marker and a whirl-like appearance (4). The diagnosis of gossypiboma is difficult using only radiographs; however, combined use with ultrasonography can occasionally enable detection of retained sponges. Previously reported ultrasonographic appearances of gossypibomas include: (i) echogenic area with intense acoustic shadow, as seen in our case; (ii) well-circumscribed cystic mass containing distinct internal hyperechoic, wavy, striped focus; and (iii) nonspecific pattern, simulating complex masses (4, 10). In this patient, ultrasonography revealed a strong acoustic shadow around a round-shaped mass in which connectivity to the surrounding organs was not observed. These diagnostic characteristics were consistent with the features of a retained surgical sponge (11). Further diagnostic tool, PET/CT, can be used to determine the metastatic state and characteristics of such a mass. In this case, the PET/CT fusion image showed circular and rim-shaped ^18^F-FDG uptake in the abdominal mass without any uptake in the center. This feature was consistent with the PET/CT characteristics of gossypibomas reported in the human literature (12).

In humans, ^18^F-FDG PET/CT is regarded as a useful imaging modality for evaluating tumor metastasis (13). In addition, it can be clinically utilized when suspecting various infectious and inflammatory conditions owing to the nonspecific nature of ^18^F-FDG accumulation. In human medicine, the commonly reported ^18^F-FDG PET/CT feature of gossypiboma was low central uptake, with high peripheral uptake accumulation corresponding to an active inflammatory reaction near the fibrotic capsule. The findings of a radiopaque marker within the internal soft tissue mass have also been described (12). In this case, the maximum and mean SUV of the abdominal mass were 4.747 and 2.590, respectively, which had higher ^18^F-FDG uptake than the other abdominal organs, indicating inflammation or metastasis. The PET/CT findings revealed an abdominal mass with low central uptake. Moreover, this combined PET/CT image revealed an internal radiopaque mark. The peripheral region of the mass had a high level of ^18^F-FDG uptake due to the fibroblastic content. These features were consistent with the PET/CT characteristics of gossypibomas that had been reported in human medical literature (8, 14). Therefore, it was possible to suspect gossypiboma in this dog. However, a diagnosis could not be made based on the uptake characteristics of ^18^F-FDG alone.

Increased FDG uptake on PET/CT can be seen in patients not only with gossypibomas due to their foreign body reaction around the retained surgical gauze, but also with tumors. In medical literature pertaining to humans, some case reports have demonstrated that gossypibomas could produce a false-positive PET/CT image by mimicking tumors in various locations such as the abdomen, thorax, and neck (12). False positives arise because some inflammatory or infectious processes absorb FDG at rates similar to those of uptake by tumor tissues (5). The central region of a tumor with significant central necrosis or bleeding can exhibit an absence of ^18^F-FDG uptake on PET/CT in humans (8). This pattern is similar to the PET/CT features shown in our patient, which might produce a false-positive result on combined PET/CT scanning of a neoplasm. Gossypiboma should be considered in the differential diagnosis of abdominal masses if high peripheral ^18^F-FDG uptake and lower uptake of the center can be observed on PET/CT, particularly when the patient has a history of previous abdominal surgery. Therefore, gossypiboma diagnosis based only on PET/CT images has some limitations, and further imaging tools, including radiography and ultrasonography, should be studied for making an appropriate diagnosis of gossypibomas.

In conclusion, this case report demonstrates the characteristics of PET/CT imaging in a dog with an abdominal gossypiboma. To our best knowledge, abdominal gossypiboma in a canine identified using ^18^F-FDG PET/CT has not been reported in the veterinary literature. PET/CT examination revealed low central uptake and high peripheral uptake, corresponding to an active inflammatory reaction near the fibrotic capsule, in a dog with gossypiboma. These findings should be interpreted with caution and raise suspicion of gossypibomas, as shown in this case.

## Data availability statement

The original contributions presented in the study are included in the article/supplementary material, further inquiries can be directed to the corresponding author.

## Ethics statement

Ethical review and approval was not required for the animal study because this is the case report of a clinical patient, not a experimental research paper on animals. Written informed consent was obtained from the owners for the participation of their animals in this study. Written informed consent was obtained from the owner of the patient for the publication of this case report. The authors would like to extend their appreciation for this consent.

## Author contributions

S-HK and SL interpreted the patient data and wrote the manuscript. Both authors have approved this manuscript.

## References

[1] Batra R Gautam R Manchanda A Ghuliani D. A case of two abdominal gossypibomas in a patient: a rare case report. J Gastrointest Abdom Rad. (2021) 4:161–5. 10.1055/s-0041-1723925

[2] Louvet A Duconseille AC. Imaging diagnosis—ultrasound uncommon features of an abdominal gossypiboma in a dog. Vet Radiol Ultrasound. (2017) 58:E68–70. 10.1111/vru.12453 27866380

[3] Auger M Olin S Morandi F. Novel CT features of an abdominal gossypiboma in a female dog. Case Rep Vet Medicine. (2019) 24:2865484. 10.1155/2019/2865484 31341697 PMC6613033

[4] Yuh-Feng T Chin-Chu W Cheng-Tau S Min-Tsung T. FDG PET CT features of an intraabdominal gossypiboma. Clin Nucl Med. (2005) 30:561–3. 10.1097/01.rlu.0000170227.56173.2f 16024957

[5] Ghersin E Keidar Z Brook OR Amendola MA Engel A. A new pitfall on abdominal PET/CT: a retained surgical sponge. J Comput Assist Tomogr. (2004) 28:839–41. 10.1097/00004728-200411000-00019 15538161

[6] Zantvoord Y van der Weiden RM van Hooff MH. Transmural migration of retained surgical sponges: a systematic review. Obstet Gynecol Surv. (2008) 63:465–71. 10.1097/OGX.0b013e318173538e 18559122

[7] Kumar GS Ramani S Mahajan A Jain N Sequeira R Thakur M. Imaging of retained surgical items: a pictorial review including new innovations. Indian J Radiol Imaging. (2017) 27:354–61. 29089689 10.4103/ijri.IJRI_31_17 PMC5644334

[8] O'connor AR Coakley FV Meng MV Eberhardt SC. Imaging of retained surgical sponges in the abdomen and pelvis. AJR Am J Roentgenol. (2003) 180:481–9. 10.2214/ajr.180.2.1800481 12540456

[9] Abdul-Karim FW Benevenia J Pathria MN Makley JT. Case report 736: retained surgical sponge (gossypiboma) with a foreign body reaction and remote and organizing hematoma. Skelet Radiol. (1992) 21:466–9. 10.1007/BF00243075 1439900

[10] Merlo M Lamb CR. Radiographic and ultrasonographic features of retained surgical sponge in eight dogs. Vet Radiol Ultrasound. (2000) 41:279–83. 10.1111/j.1740-8261.2000.tb01491.x 10850880

[11] Sugano S Suzuki T Iinuma M Mizugami H Kagesawa M Ozawa K. Gossypiboma: diagnosis with ultrasonography. J Clin Ultrasound. (1993) 21:289–92. 10.1002/jcu.1870210415 8478466

[12] Kuruva M Jambhekar K Viswamitra S Ram R. Gossypiboma of axilla: imaging pitfalls on fluorodeoxyglucose positron emission tomography and computed tomography. Indian J Nucl Med. (2018) 33:143. 10.4103/ijnm.IJNM_140_17 29643677 PMC5883434

[13] Özaydin I Elmali M Çelik B Kocabicak D. Intrathoracic gossypiboma: a diagnostic challenge. Diagn Interven Radiol. (2013) 19:263. 10.5152/dir.2013.172 23439257

[14] de Llanos CG Navarro PC Gilart JF Suárez PR Serhald MH Saavedra TR. Intrathoracic gossypiboma interpreted as bronchogenic carcinoma. another false positive with positron emission tomography. Arch Bronconeumol. (2007) 43:292–4. 10.1016/S1579-2129(07)60070-6 17519142

