# Corrigendum: Case report: ^18^F-2-deoxy-2-fluoro-D-glucose positron emission tomography/computed tomography image findings of a dog with gossypiboma

**DOI:** 10.3389/fvets.2024.1512990

**Published:** 2024-10-31

**Authors:** Su-Hyeon Kim, Sungin Lee

**Affiliations:** Department of Veterinary Surgery, College of Veterinary Medicine, Chungbuk National University, Cheongju-si, Chungbuk, Republic of Korea

**Keywords:** gossypiboma, ^18^F-deoxy-2-D-glucose, positron emission tomography/computed tomography, dog, canine

In the published article, there was an error. Two specific sections in the Abstract and Case description may cause misunderstandings regarding the referral process of the patient.

A correction has been made to **Abstract**, paragraph 1. This sentence previously stated:

“A 13-year-old, spayed, female mixed breed dog that had previously undergone mastectomy and ovariohysterectomy was referred for evaluation of metastasis after surgery.”

The corrected sentence appears below:

“A 13-year-old, spayed, female mixed breed dog that had previously undergone mastectomy and ovariohysterectomy at our institution was referred to the nuclear medicine department for metastasis evaluation following surgery.”

A correction has been made to **Case description**, paragraph 1. This sentence previously stated:

“A 13-year-old, spayed, female mixed breed dog was referred to us for evaluation of metastasis.”

The corrected sentence appears below:

“A 13-year-old, spayed, female mixed breed dog was referred initially to the nuclear medicine department for metastasis evaluation after surgery at our institution.”

The authors apologize for this error and state that this does not change the scientific conclusions of the article in any way. The original article has been updated.

